# Natural biomass derived hard carbon and activated carbons as electrochemical supercapacitor electrodes

**DOI:** 10.1038/s41598-019-52006-x

**Published:** 2019-11-08

**Authors:** Sourav Ghosh, Ravichandran Santhosh, Sofia Jeniffer, Vimala Raghavan, George Jacob, Katchala Nanaji, Pratap Kollu, Soon Kwan Jeong, Andrews Nirmala Grace

**Affiliations:** 1 Centre for Nanotechnology Research, Vellore Institute of Technology, Vellore, Tamil Nadu India; 2 Centre for Nano Materials, International Advanced Research Centre for Powder Metallurgy and New Materials, Hyderabad, 500005 Telangana India; 3 CASEST, School of Physics, University of Hyderabad, Hyderabad, Telangana 500046 India; 4 Climate Change Technology Research Division, Korea Institute of Energy Research, Yuseong-gu, Daejeon 305-343 South Korea; 5 Department of Metallurgical and Materials Engineering, Indian Institute of Technology Madras, Chennai, 600036 Tamil Nadu India; 6 Department of Chemistry, Indian Institute of Technology Madras, Chennai, 600036 Tamil Nadu India

**Keywords:** Porous materials, Structural properties, Porous materials, Porous materials, Porous materials

## Abstract

With every moving day, the aspect that is going to be the most important for modern science and technology is the means to supply sufficient energy for all the scientific applications. As the resource of fossil fuel is draining out fast, an alternative is always required to satisfy the needs of the future world. Limited resources also force to innovate something that can utilise the resource more efficiently. This work is based on a simple synthesis route of biomass derived hard carbon and to exploring the possibility of using it as electrochemical supercapacitors. A cheap, eco-friendly and easily synthesized carbon material is utilized as electrode for electrochemical energy-storage. Four different hard carbons were synthesized from KOH activated banana stem (KHC), phosphoric acid treated banana stem derived carbons (PHC), corn-cob derived hard carbon (CHC) and potato starch derived hard carbons (SHC) and tested as supercapacitor electrodes. KOH-activated hard carbon has provided 479.23 F/g specific capacitance as calculated from its cycle voltammograms. A detailed analysis is done to correlate the results obtained with the material property. Overall, this work provides an in depth analysis of the science behind the components of an electrochemical energy-storage system as well as why the different characterization techniques are required to assess the quality and reliability of the material for electrochemical supercapacitor applications.

## Introduction

The market for small portable electronics and hybrid electric devices is fast-growing thereby demanding an immediate supply of developed storage systems of electrochemical energy. In this regard, lithium ion batteries (LIBs) and electrochemical supercapacitors (SCs) have recently gathered a huge attention. It is quite well known that both the devices have their set of advantages and drawbacks. Electrochemical supercapacitors possess high power density with respect to batteries but suffer from poor energy density. Whereas batteries possess high energy density to that of supercapacitors with low power density. However the power density exhibited by supercapacitors is less compared to conventional capacitors but with improved energy density^1,2^. A rapid charge storage mechanism is observed in case of supercapacitors, attributing to their decreased charging time, improved cyclability and thereby capacitance^3^. With careful selection of electrode material and with the use of simple, cost effective synthesis techniques, supercapacitors can be developed for commercial applications on a larger scale. The most critical component in a supercapacitor is the electrode material used, which determines the ultimate performance of the fabricated supercapacitor. Thus significant research in exploring new electrode materials to obtain improved performance is being progressed. Typically supercapacitors can be classified into three types based on the mechanisms of charge storage: (1) Electrochemical double layer supercapacitors (EDLC),(2) Pseudocapacitors and (3) hybrid supercapacitos^3,4^. In EDLC based supercapacitors the charges are stored based on the double layer formation happening at the electrode surface. The surface area of the electrode material used is of utmost importance in this type of supercapacitor^3,5^. It is well known that carbonaceous materials possess highest surface area compareg to metal oxides/sulfides/phosphides^6–9^. Thus various carbonaceous materials such as activated carbon (AC)^3^, graphene^10^, metal carbide derived carbon^11^, ordered mesoporous carbon^12,13^, carbon aerogels^14,15^ and carbon composites^16–18^ have been tested for their supercapacitive behavior. Among the carbonaceous materials used in literature, ACs has outstood the others. Activated carbons usually possess higher porosity, high chemical and thermal stability, surface area and packing density^19,20^. Activated carbons employed as electrodes for supercapacitors are usually obtained from renewable resources, biowastes, sawdust, neem leaves, coconut shell, bamboo, see weeds etc^21–30^.

Though carbon is widely used, they still face a major drawback of poor specific capacitance compared to that provided by conducting polymers^31^ and metal oxides^32,33^ as Faradaic reactions occur in these materials. However, carbon is the currently used material in the fabrication of commercial supercapacitors due to its low cost and high conductivity^34–42^. Among the carbonaceous materials indicated previously, AC is the widely used material for commercial applications^43–45^. Activated carbons are usually synthesized from different types of biomass such as pitch, coal, tea leaves, coconut shells, cellulose, polymers etc. A type of disordered carbon material, which is popularly known as hard carbon has been studied intensively. Hard carbon is a terminology typically used to distinguish carbons that are non-graphitizable from graphitizable by heat treatment^46,47^. Hard carbons are basically non-graphitizable carbons with amorphous nature^4^, whereas soft carbons can be graphitized by introducing it to heat treatment. Hard carbons can be obtained by carbonization of a huge range of several precursors like thermo-setting polymers, cellulose, coconut shell, corn, charcoal, etc.^48^, out of which biomass deserves more scientific interest. Biomass has been extensively investigated as a carbonaceous precursor due to its abundance, low cost, easy availability compared to other carbon sources and these could be easily converted to conductive carbon, which would be of low cost and an efficient way for recycling biomasses. Though high capacitance has been achieved with certain carbon materials like CNTs and graphene, these materials are highly expensive due to the intricate preparation processes and insufficient raw materials, which greatly limit the possibility of large scale applications. In this regard, it is highly necessary to synthesize low cost carbon materials from biomass-based sources which is compared in Table 1.

**Table 1:** Comparison of different electrode materials (from literature) with the prepared biomass derived materials (this study).

Electrode material	Specific capacitance	Electrolyte
PMF resin based AC^40^	210 Fg^−1^ @2 mVs^−1^	6 M KOH
Waste hemp fibres derived AC^61^	122 Fg^−1^ @5 mVs^−1^	1 M H_2_SO_4_
Nomax derived AC^62^	175 Fg^−1^ @5 mVs^−1^	5.25 M H_2_SO_4_
PAN derived AC fibre by ameliorative chemical activation^63^	158 Fg^−1^ @5 mVs^−1^	1 M H_2_SO_4_
Lignin derived AC^64^	102.3 Fg^−1^ @1 mVs^−1^	6 M KOH
Lignosulphonate-cellulose derived AC^50^	246 Fg^−1^ @2 mVs^−1^	6 M KOH
Rotten carrot derived AC^34^	137 Fg^−1^ @10 mVs^−1^	6 M KOH
Rice husk derived AC^65^	143 Fg^−1^ @5 mVs^−1^	6 M KOH
Rice husk derived NaOH activated AC^36^	172.3 Fg^−1^ @5 mVs^−1^	0.5 M K_2_SO_4_
Compact disk derived AC^66^	51 Fg^−1^ @10 mVs^−1^	EMIMBF_4_
Steam activated corncob derived carbon^67^	298 Fg^−1^ @10 mVs^−1^	6 M KOH
Waste tire derived AC^68^	106.4 Fg^−1^ @2 mVs^−1^	6 M KOH
KHC	479.23 Fg^−1^ @1 mVs^−1^	6 M KOH
CHC	309.81 Fg^−1^ @2 mVs^−1^	6 M KOH
PHC	202.11 Fg^−1^ @2 mVs^−1^	6 M KOH
SHC	99.9 Fg^−1^ @2 mVs^−1^	6 M KOH

**Figure 1:**
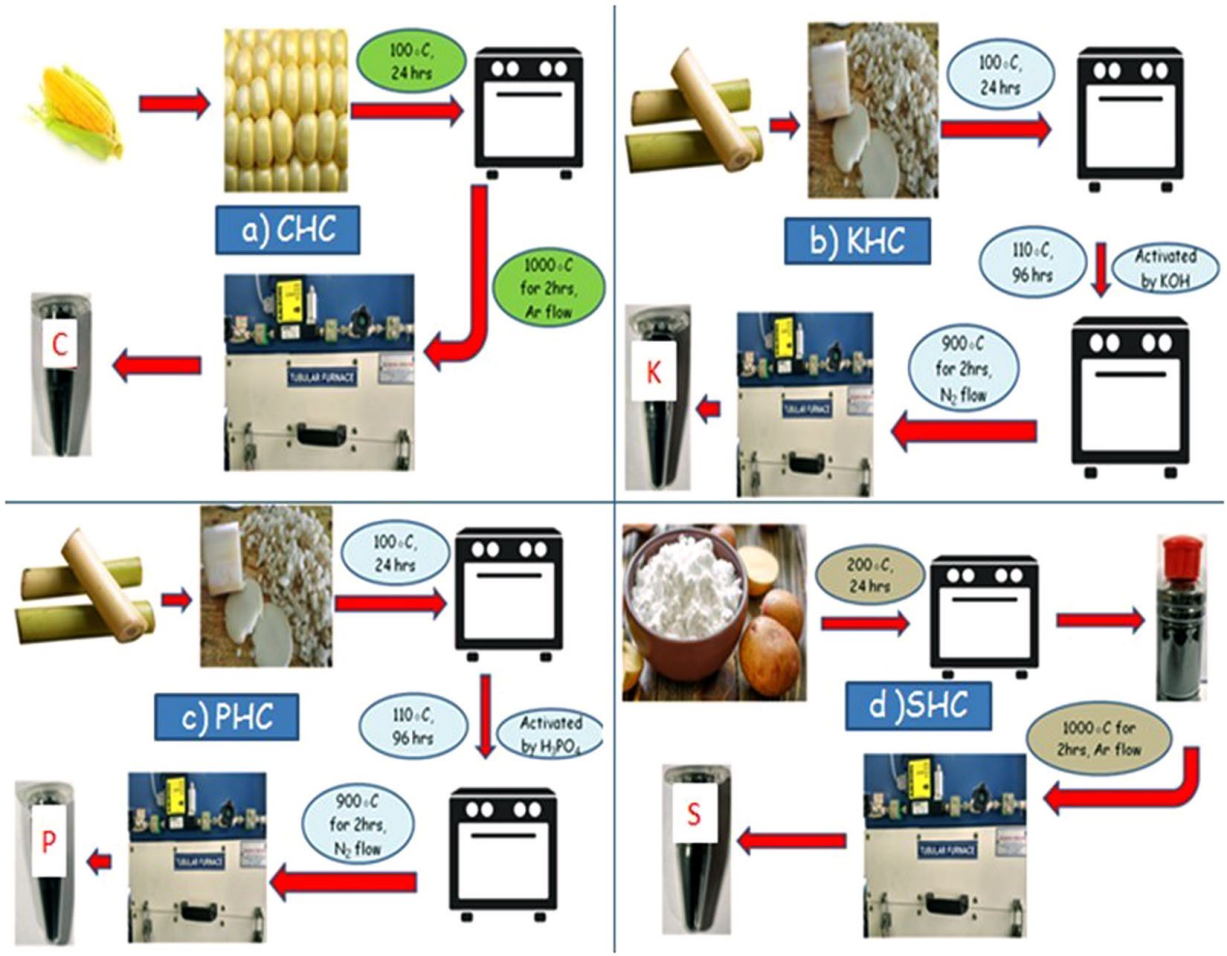
A pictorial representation of the synthesis procedure of hard carbon and activated carbon materials (**a**) CHC (b) KHC (**c**) PHC and (**d**) SHC.

In the present work, hard carbons were prepared from different biomass viz. corn cob, banana stems and starch, and investigated for the possibilities of utilising those synthesized hard carbons for electrochemical supercapacitor electrodes (Fig. 1). Corn cob is an abundantly-present, agricultural by-product contained with high amount of cellulose and hemicelluloses like banana stems. Cellulose being cheap and abundantly available makes them appropriate candidate for the synthesis of carbon materials in the place of resin based precursors^49,50^. Results showed that the KOH activated hard carbon derived from banana stem have a capacitance value of 479.23 F/g. Interestingly, the corn cob derived hard carbon also gave a maximum value of capacitance of 309.81 F/g under the same conditions.

## Results and Discussions

### Structural and morphological characterizations

**Figure 2:**
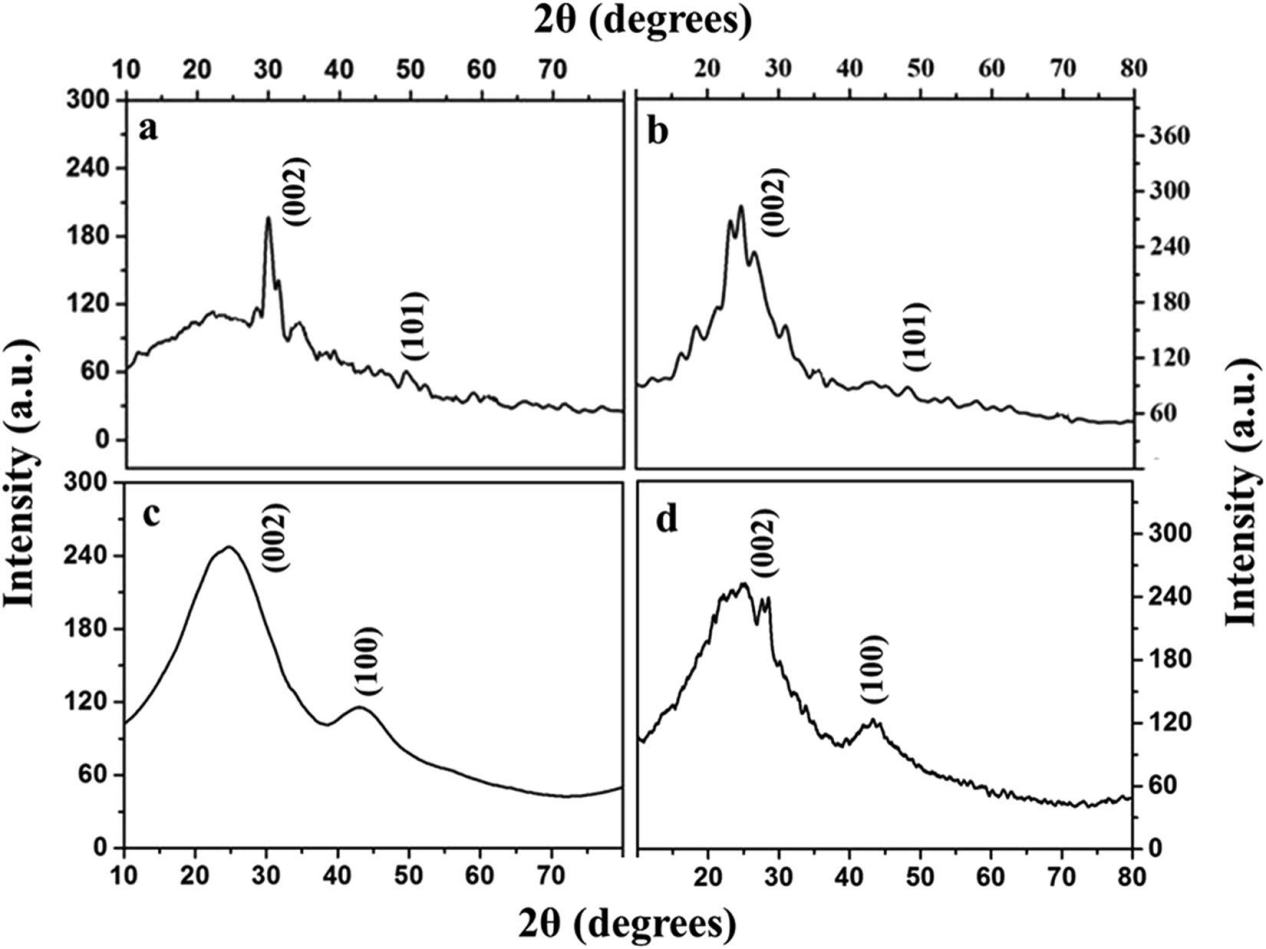
Powder X-Ray diffraction spectrum of all the four carbon samples (**a**) KHC (**b**) PHC (**c**) CHC and (**d**) SHC.

The X-ray diffraction spectrum of four different carbon materials derived from diverse sources viz. KHC, PHC, CHC and SHC are given in Fig. 2. All the samples present two broad reflection peaks around 22–25° and 43–45° typical of highly disordered diffracting planes i.e. the distance between graphene sheets and the second broad peak correspond to (100) diffraction planes. In Fig. 2a, the peaks at 24.28°, 44.43° and 79° are correlated to the (002), (100) and (110) diffraction peaks. Similar peaks showing the formation of hard carbon were also observed in other cases^51^. The pattern of the peaks is so typical of non-graphitic carbon materials with highly disordered nano-crystalline structure, which is a proof of hard carbon. The broad peak ensures the amorphous nature of these carbon materials. In the case of KHC (Fig. 2a), apart from the main peaks around 23° and 44°, other minor peaks are attributed to potassium compounds happened during activation process, which is in accordance with the earlier report^39^. The crystallite size was calculated from Scherer formula and it was found that for corncob derived hard carbon material i.e. CHC has the lowest with 0.591 nm, for starch (SHC) and H_3_PO_4_ (PHC) activated hard carbon, it was calculated to be 0.627 nm and 0.639 nm respectively. However, largest of four samples in terms of crystallite size is KOH activated hard carbon with 6.09 nm (KHC). From the 2θ degree of the (002) peak, the value of interlayer distance (d_002_) is calculated to be larger than graphite (0.335 nm). The interlayer distance of hard carbon samples are 0.371 nm, 0.361 nm, 0.383 nm, 0.389 nm for corncobs derived (CHC), potato starch derived (SHC), H_3_PO_4_ activated (PHC) and KOH activated (KHC) hard carbon respectively. These interplanar distances are higher than the graphitic interplanar spacing for carbon materials and as observed, KHC derived carbon showed a larger interlayer distance and such larger interlayer distance will facilitate easier penetration of K^+^ electrolyte ions, which further will increase the capacitance characteristics.

**Figure 3:**
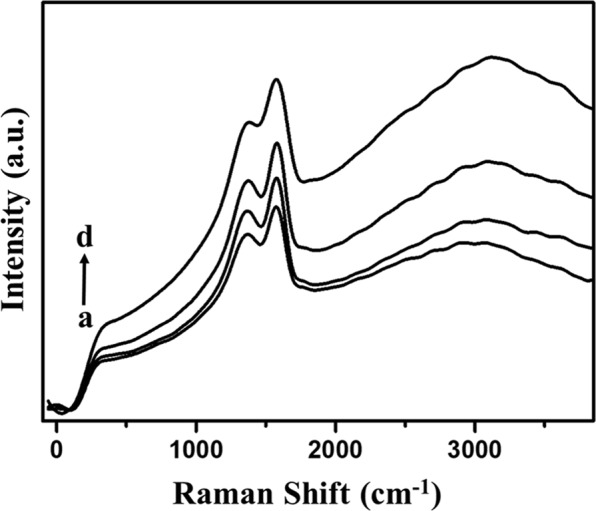
FT-Raman Spectroscopy of all the four carbon samples (**a**) PHC (**b**) CHC (**c**) SHC and (**d**) KHC.

Raman spectroscopy is very useful to determine the structure and defects as well as the disordered nature of carbon materials (Fig. 3). Raman shift could be seen around 1310 to 1350 cm^−1^ for disorder or defect peak of D band and nearby 1580 cm^−1^ for graphitic peak of G band as mentioned in various literatures. Graphitic peak is observed because of the E_2g_ mode of vibration and relevant stretching of the C-C carbon bond, while the D band peak is due to A_1g_ mode of vibration. Corncob, starch derived hard carbon materials produces peaks at 1348 and 1340 cm^−1^(D band) and at 1582 and 1564 cm^−1^ (G band) respectively. In banana stem samples; KOH and H_3_PO_4_ activated carbons produced peaks at 1335 and 1334 cm^−1^ for D band and 1583 and 1580 cm^−1^ for G band. Partial graphitization can be confirmed by the G-band peak present for all the four samples^52^. Moreover, from this, the intensity ratio of different peaks can give a detailed look into the disordered nature of the hard carbon samples. The ratio of intensity of D band (I_D_) to the intensity of G band (I_G_) for corncob, starch, KOH activated banana stem and H_3_PO_4_ activated banana stem samples are 1.014, 1.048, 1.095, and 1.074 respectively. The intensity ratios are higher than the standard value of carbon I_D_/I_G_ ratio where it should be equal to zero for non-defective carbon material. Symmetrical disturbance in nano or microscale can cause this type of disorderness. This again shows the disordered nature of all the hard carbon samples. The KOH-activated hard carbon sample has more disordered nature, which is more useful for energy storage applications like supercapacitors due to the presence of active sites.

### Morphological characterization

**Figure 4:**
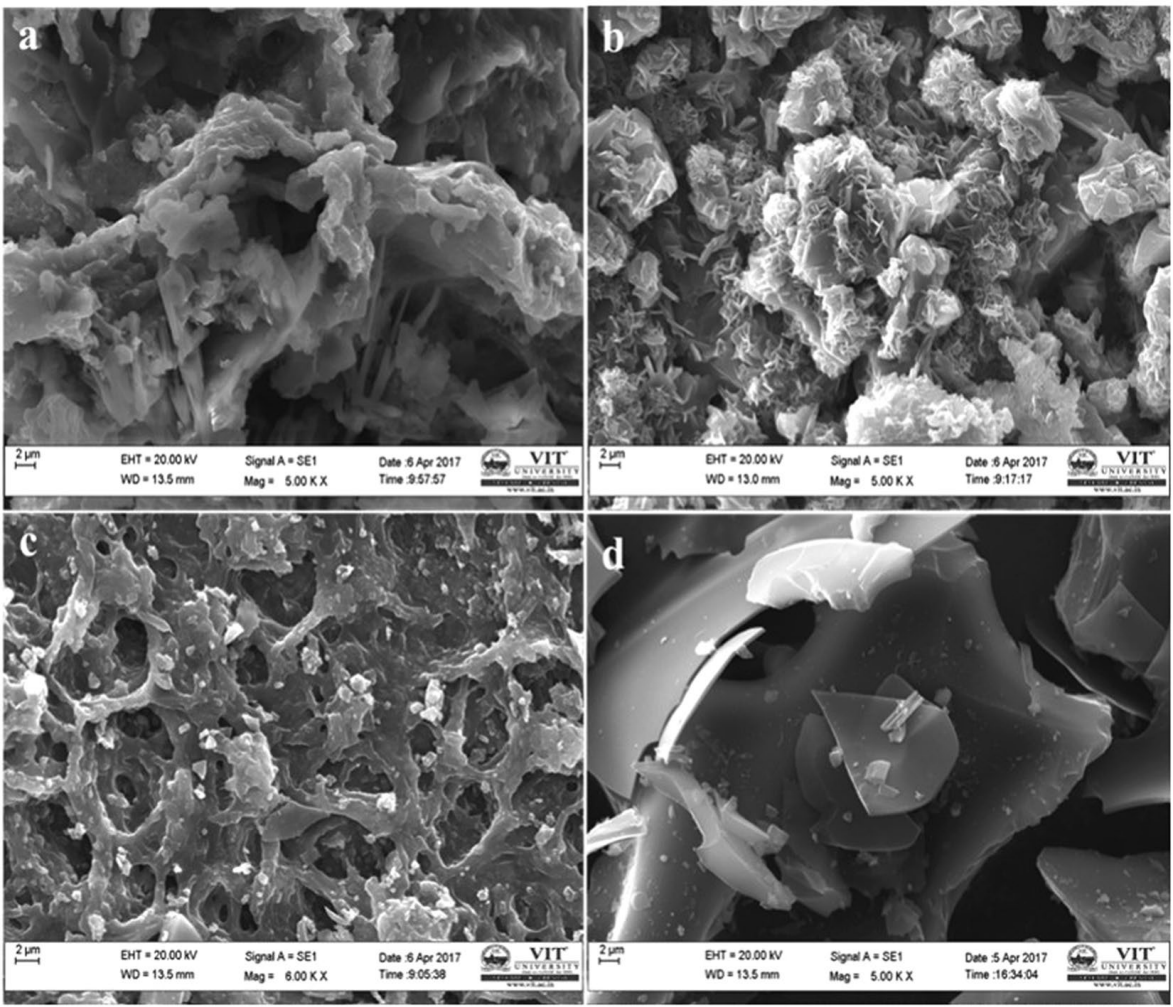
FE-SEM images of all the four carbon samples (**a**) KHC (**b**) PHC (**c**) CHC and (**d**) SHC.

**Figure 5:**
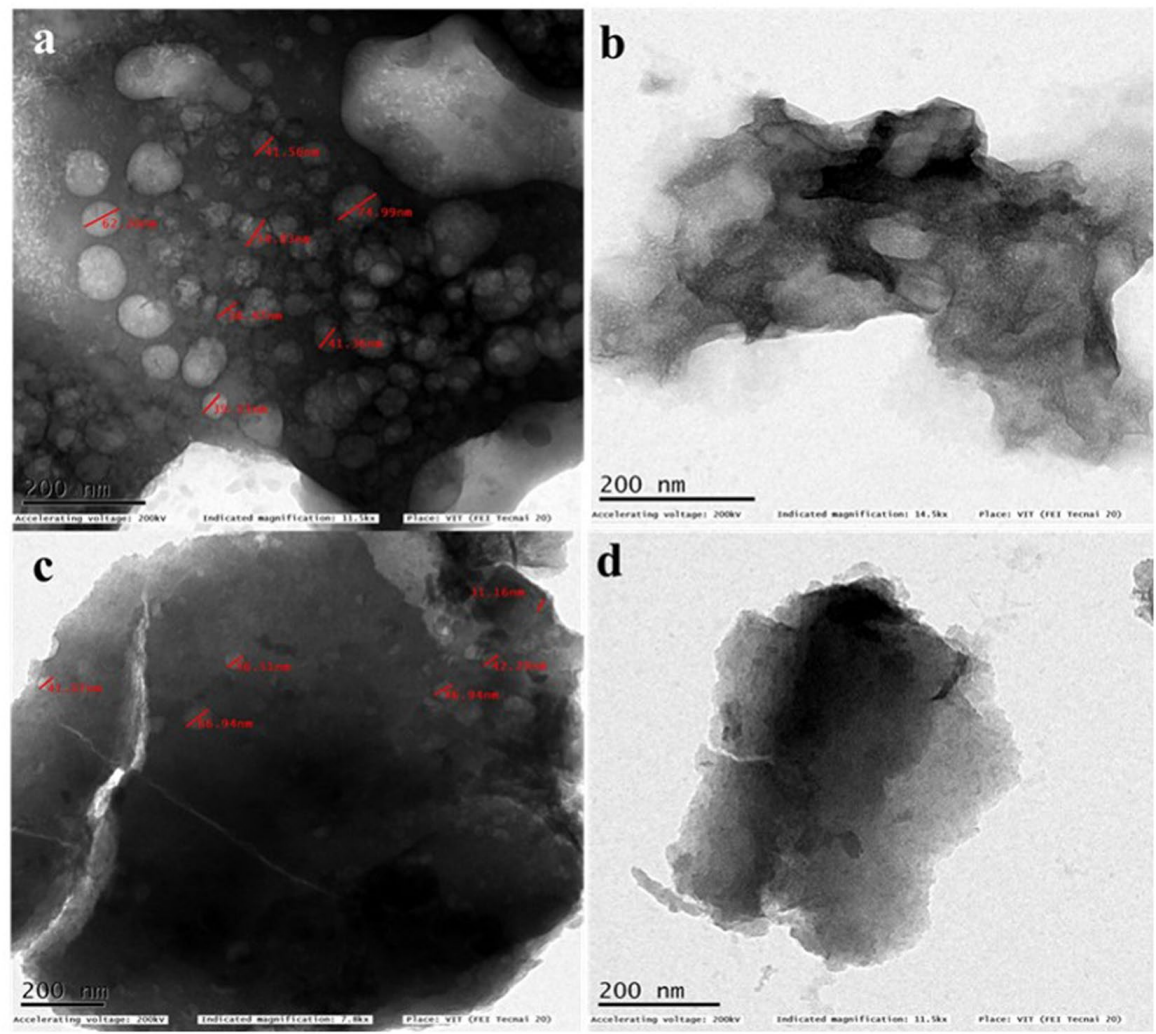
TEM images of all the four carbon samples (**a**) KHC (**b**) PHC (**c**) CHC and (**d**) SHC.

The morphological feature of the samples as observed by SEM analysis is given in Fig. 4. The potato starch derived carbon (SHC) did not show any porosity as observed from SEM images (Fig. 4d). The porous nature of material could be advantageous in capacitor applications than the non-porous material. So, from the SEM images, it can be said that SHC might be less advantageous than the other samples. Disordered and uneven structures were observed in all the images. SHC has shown flake kind of structures, where KHC and PHC have shown totally uneven morphological characteristics. SEM image of CHC in Fig. 4c has shown more porous structure of all the materials visibly. To ascertain the pore volume, BET analysis has been done and analysed in the later part of this paper. It should be noted that all kinds of pores may not be helpful for capacitor applications. Mesopores and micropores are reported to be more helpful for charge transfer and hence give better performance as supercapacitors or ultracapacitors. High Resolution TEM images as shown in Fig. 5 are also in good agreement with SEM images, which further confirmed the porosity of samples. Micro range crystallites might be formed by various short-range structures of carbon layers arranged in parallel in the increasing order of the rise in the temperature required for carbonization. This phenomenon occurs due to the reduction of few defects of the material and corresponding carbon atoms rearrangement process. The diameter of the pores is in nanometre range except SHC, which is evidently non-porous hard carbon sample.

### N_2_ adsorption and desorption isotherm analysis

**Figure 6:**
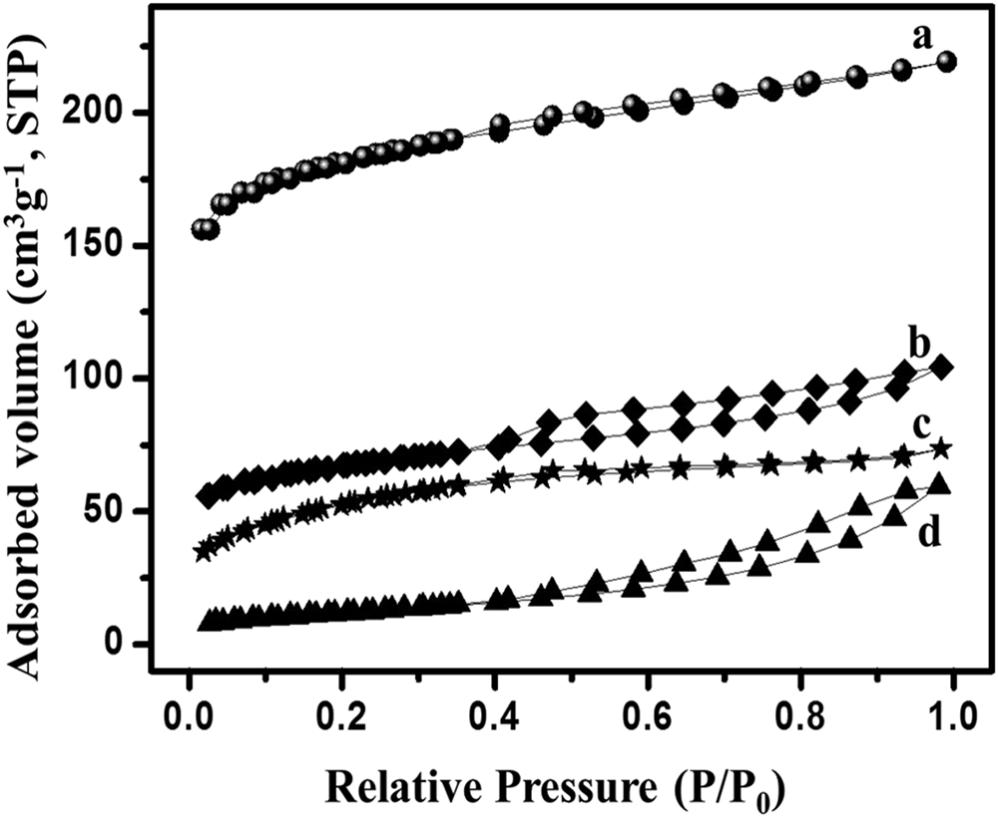
N_2_-sorption isotherm analysis for all four carbon samples (**a**) KHC (**b**) CHC (**c**) SHC and (**d**) PHC.

To further know its porous nature, BET analysis of the samples was done and the results are given in Fig. 6. The typical curve of this graph resembles a type IV graph of isotherm analysis according to the classification by IUPAC. The sharp rise in the curve before 0.45 P/P_0_ region denotes the possibility of micropores in the KHC sample (Fig. 6a). The loop of hysteresis in higher relative pressure zone is an indicative of mesopores of developed size and the very short tail on the zone of 1.0 P/P_0_ might be due to the presence of very less amount of macropores. Although micropores and mesopores are more desirable for electrochemical energy storage applications, the presence of macropores in such less numbers can also be advantageous. Macropores can contribute as the transport zone in the inner network of carbon material even though it has minimal contribution in increasing surface area of the material^3,53^. Figure 6d depicts the isotherm curve of PHC i.e. H_3_PO_4_ activated hard carbon. The low slope in the lower zone of this curve ensures the presence of micropores, which is negligible in this material. The curve shows a typical Type IV curve along with H_3_ hysteresis loop. The positioning of this loop confirms that this material comprises of large and developed mesopores^54^. In supercapacitors all kinds of pores have significant contribution and so this material might not be promising for supercapacitor applications. When compared with PHC, CHC has slightly more number of micropores (Fig. 6b). The N_2_ adsorption and desorption curve again shows type IV curve with hysteresis loop on the higher zone of relative pressure (>0.4 P/P0)^37,55^. The material which holds this kind of curve comprises huge amount of mesopores of different size and volume. As mentioned before, CHC has more amounts of visible pores which is evident from the SEM image. Now as this isotherm analysis also proves the same, it is quite evident that the morphological analysis and the pore and surface area analysis are in agreement with each other. According to the above discussion, the type-IV curve of SHC (Fig. 6c) has shown the possibilities of non-porous material. The long tail at the end can be attributed to the highly irregular macropores in a really minimal amount. Effect of tensile strength can be a reason to the sudden drop of volume adsorption phenomenon in the range between 0.4–0.5 P/P_0_. This result also supports the morphological study of the same material. Overall, it can be said and justified that potato starch derived hard carbon (SHC) may not be as useful for supercapacitors as the other three samples.

**Figure 7:**
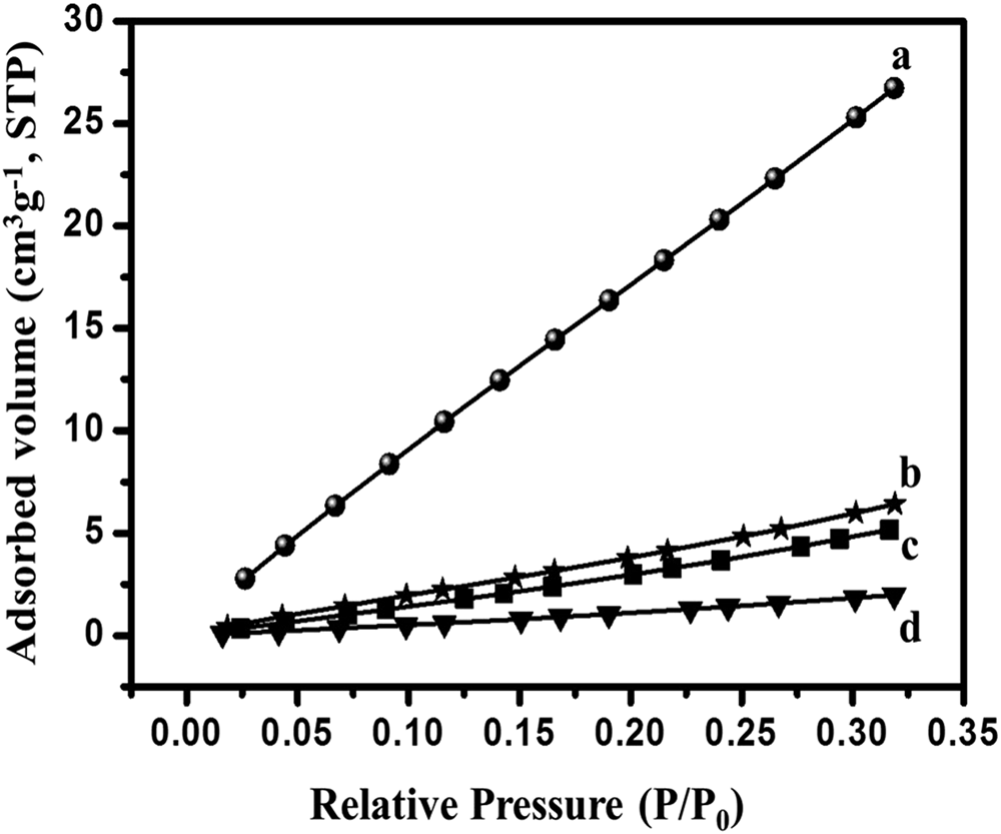
BET nitrogen adsorption/desorption isotherm surface area analysis for all four carbon samples (**a**) PHC (**b**) SHC (**c**) CHC and (**d**) KHC.

**Figure 8:**
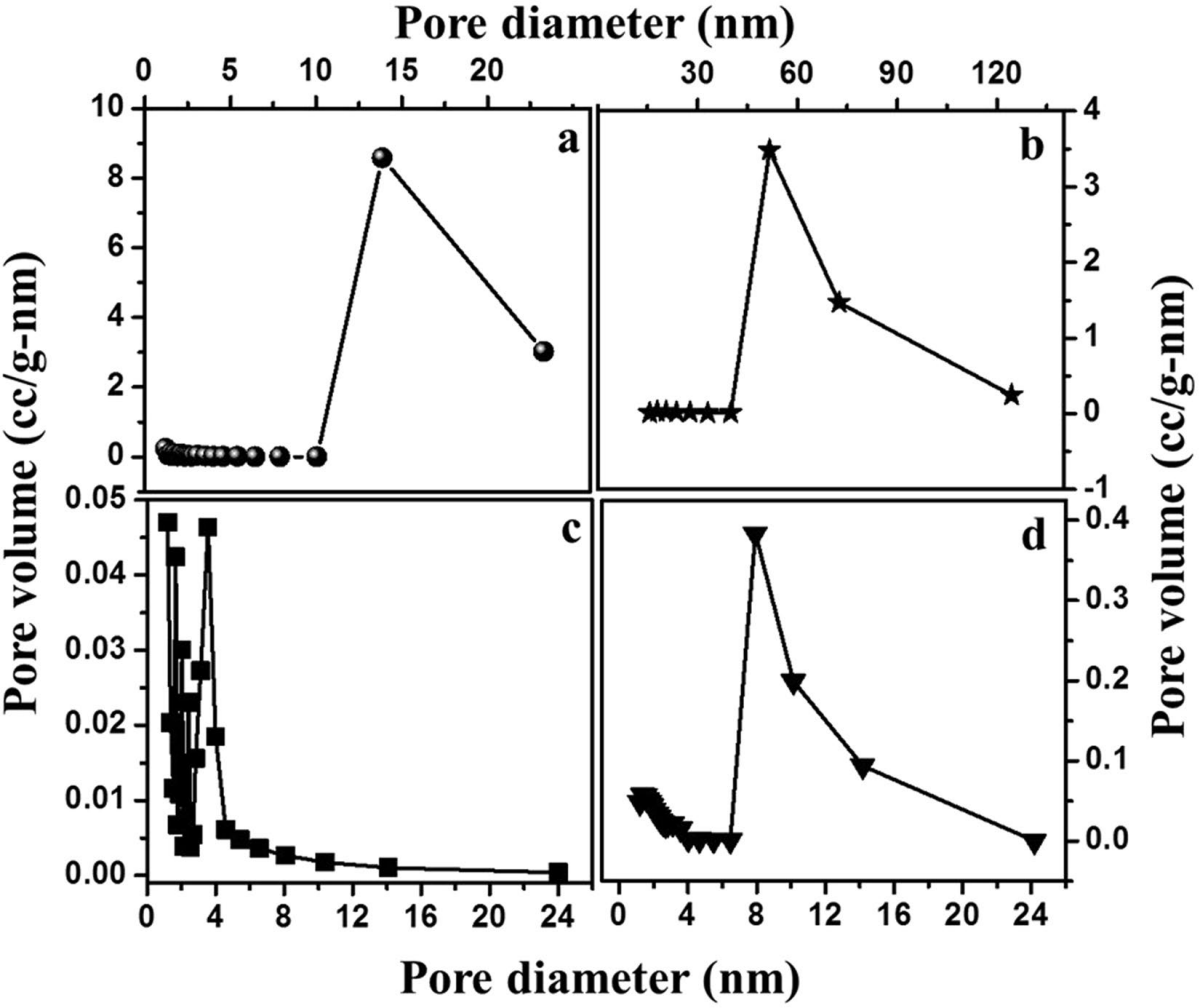
Pore size distribution of (**a**) KHC (**b**) PHC (**c**) CHC and (**d**) SHC.

BET surface plot was done to calculate the required BET surface area. All the plots are presented in Fig. 7a–d. The plot related to this surface area is in correspondence to BET. The surface area of KHC, PHC, CHC and SHC are 567.36 m²/g, 177.72 m²/g, 215.42 m²/g and 42.43 m²/g respectively. From the surface area analysis, it is quite evident that KHC and CHC have larger surface area, which may attribute for better performance of supercapacitors. Thus, KHC and CHC are better than PHC and SHC and could be suitable for storage applications. For pore size analysis, BJH analysis was done and data were calculated by de Boer method (Fig. 8a–d). The average pore diameter for KHC, PHC, CHC and SHC are 1.205 nm, 1.789 nm, 1.199 nm and 1.363 nm respectively and the corresponding average pore volume is calculated to be 0.175 cc/gm, 0.091 cc/gm, 0.107 cc/gm, 0.099 cc/gm. It is found from literature that a pore diameter close to 1 nm gives better performance as supercapacitors^3^. The value of the pore diameter for KHC is closest to 1 nm and its pore volume is also higher, which is the reason for its larger surface area. According to pore diameter and pore volume, CHC comes as second-best material to KHC as it has a diameter almost similar to KHC. Combined with the diameter, the pore volume is also higher for KHC. The higher surface area along with higher pore volume and lower diameter of pore favoured KHC as electrodes in capacitive performance.

### Electrochemical performances of hard carbon and activated carbon electrodes

**Figure 9:**
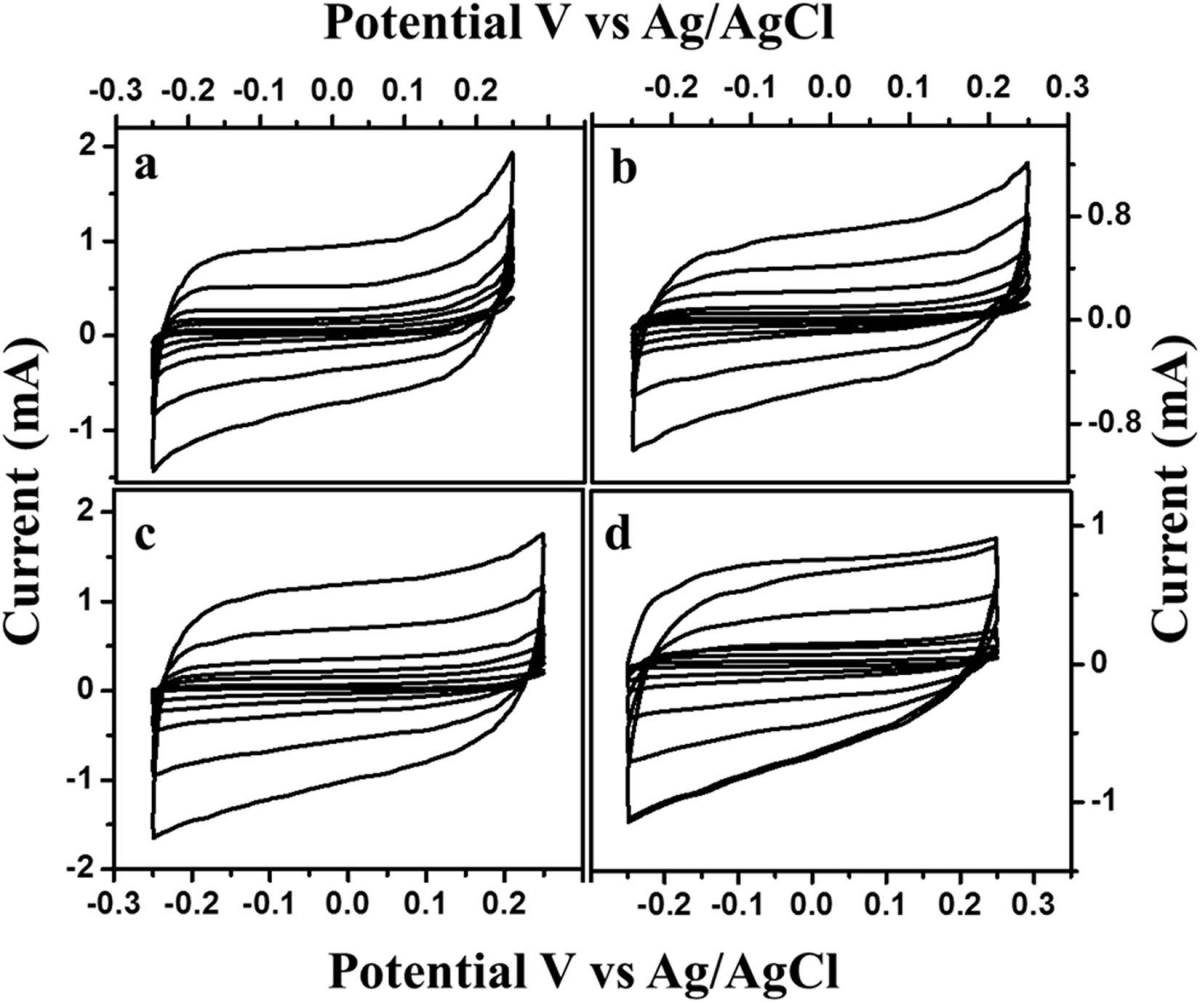
Cyclic voltammetry of different samples at different scan rates in 6 M KOH solution vs. Ag/AgCl (**a**) KHC (**b**) PHC (**c**) CHC and (**d**) SHC.

**Figure 10:**
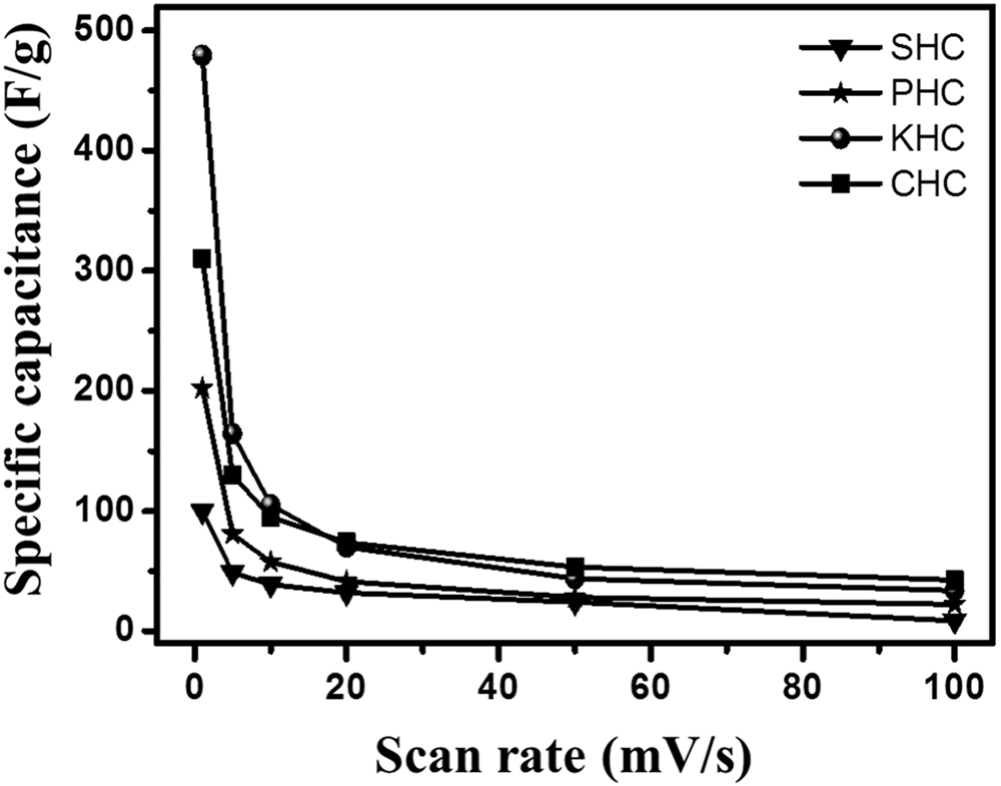
A plot of specific capacitance vs. scan rates of all the prepared hard carbon samples (**a**) CHC (**b**) KHC (**c**) PHC and (**d**) SHC.

**Figure 11:**
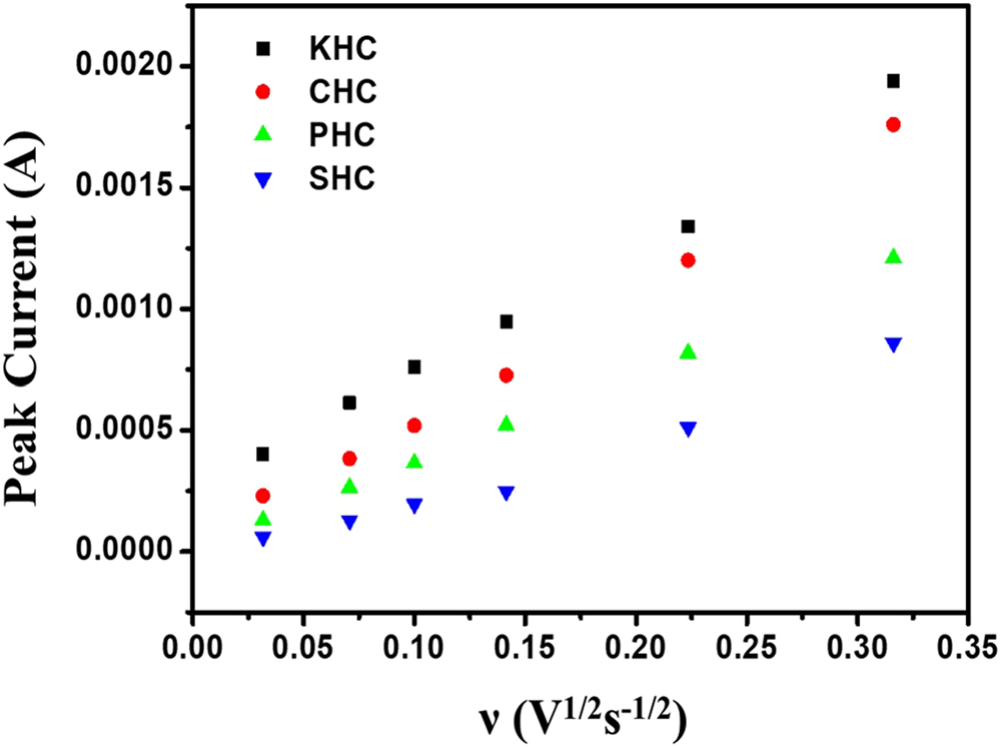
Galvanostatic charge/discharge curves of (**a**) KHC (**b**) PHC (**c**) CHC and (**d**) SHC measured at various current values in 6 M KOH solution vs. Ag/AgCl.

Electrochemical performance of the various hard carbons as electrode materials for supercapacitors was estimated using a three-electrode system in 6 M KOH solution and the typical measured curves are given in Fig. 9. From the curves, it could be seen that the cyclic voltammetry curve of KHC is particularly different from a typical rectangular behaviour of EDLCs. The presence of redox peaks are indicative of faradaic reactions, which attributes to the pseudocapacitance of the material^55^. Similar type of peaks can be seen in the other materials as well. All the materials have nearly rectangular shaped CV diagram especially Fig. 9c,d, which directs towards its double layered capacitive behaviour. Howeve r, the presence of both pseudocapacitance and double layered capacitance for activated carbon can be explained by its activation nature. As it is already clear from structural characterization of these two materials, they possess crystallinity of lower order and overall disordered structure. Probable presence of oxygen on the edge of the micro-ordered sheets inspires the hydrophilic nature in aqueous KOH solution to increase the double layered capacitive behaviour. The involvement of the same oxygen with metal ions from the electrolytic solution provides charge transfer properties that are required for pseudocapacitance. The value of capacitance increased gradually with decreased scan rates for all those materials. It might be caused due to the inability to sustain the redox reaction of many active sites, which can occur because of the lesser availability of time for hydroxyl ions to get transferred from electrolyte to the electrode surface (Fig. 10). The specific capacitance value increases from 8–13% for different materials with decreasing scan rate. KHC stands topmost with more than 13% increase in specific capacitance when the scan rate decreased from 100 mV/s to 1 mV/s. Figure 10 gives a detailed look on this behaviour of specific capacitances with respect to varying scan rates. SHC gives a low value of 99.9 F/g at 1 mV/s scan rate. PHC provides 202.11 F/g specific capacitance, which might be low compared to other two materials but not in overall sense. KHC and CHC provides a specific capacitance of 479.23 F/g and 309.81 F/g respectively. This result stays in firm agreement with all the assumption and explanations made from the different characterization techniques. High surface area, high porosity, presence of different kinds of pores and disordered structure have allowed KHC to possess active sites, which has resulted in higher value of capacitance. To understand the diffusion mechanism of the controlled electrochemical process, the same has been identified from the linearity of peak current vs square root of scan rate plot of Randles-Sevcik equation (Fig. 11). The relevant equation is,

$$\frac{I^{\prime} }{{{\rm{\upsilon }}}^{\frac{1}{2}}}=(2.69\times {10}^{5}){n}^{\frac{3}{2}}S{D}^{\frac{1}{2}}C^{\prime} $$

In the above equation, I’ is the peak current (A), ʋ is the scan rate (Vs^−1/2^), n is the number of electron lost or gained in the process, S is the surface area of the electrode (cm^2^), D is the diffusivity of the reactant (cm^2^ s^−1^) and C’ is the initial reactant concentration (mol cm^−3^)^56^. So, it is evident that the diffusivity can be understood from the slope of the plot. Thus, in terms of diffusivity the materials stand in the order of KHC > CHC > PHC > SHC. This result clearly indicates the superior performance of KHC and CHC over the other competent electrode materials.

**Figure 12:**
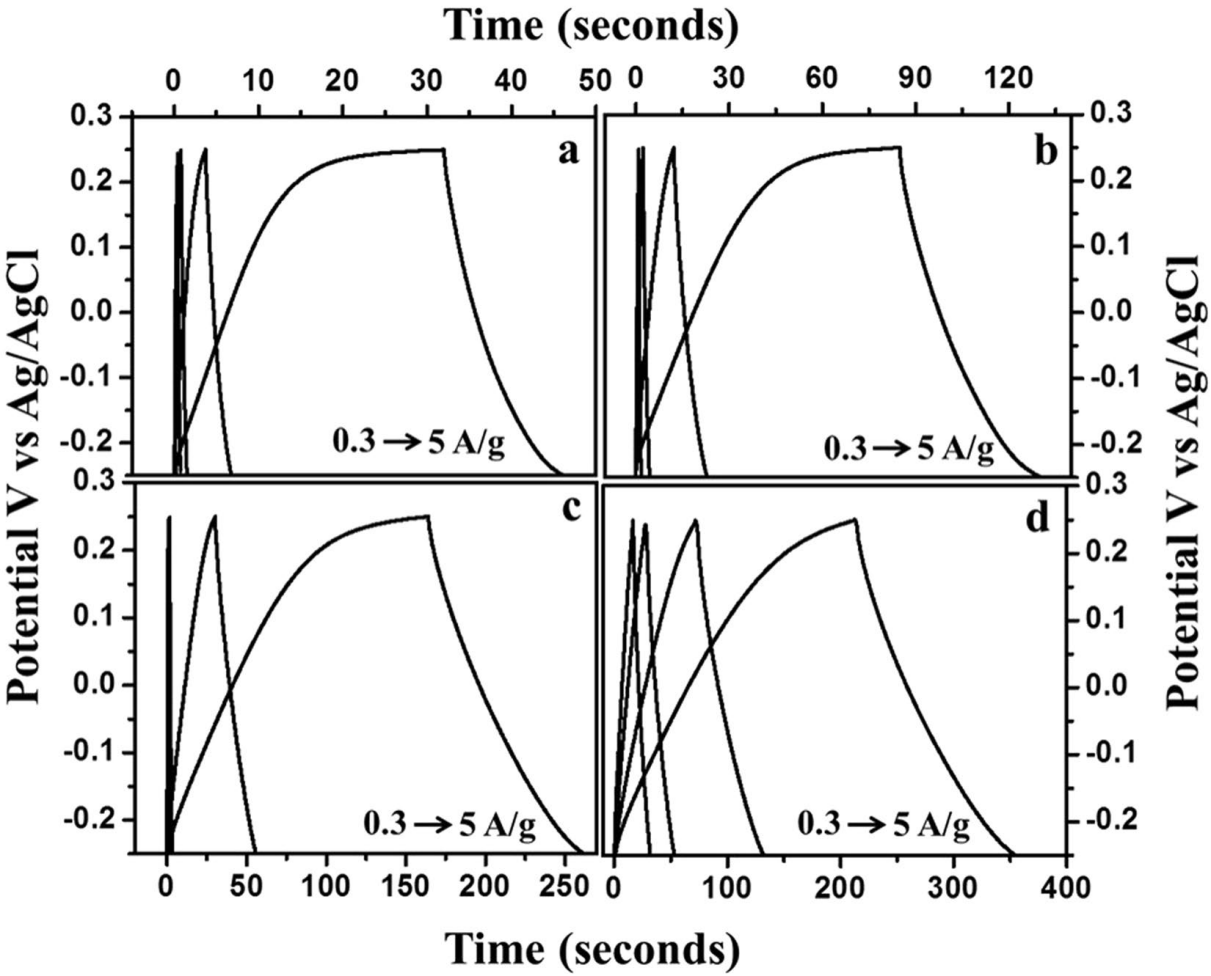
Charge discharge profile for KOH-activated hard carbons (KHC) measured at 3 mA/g current density.

**Figure 13:**
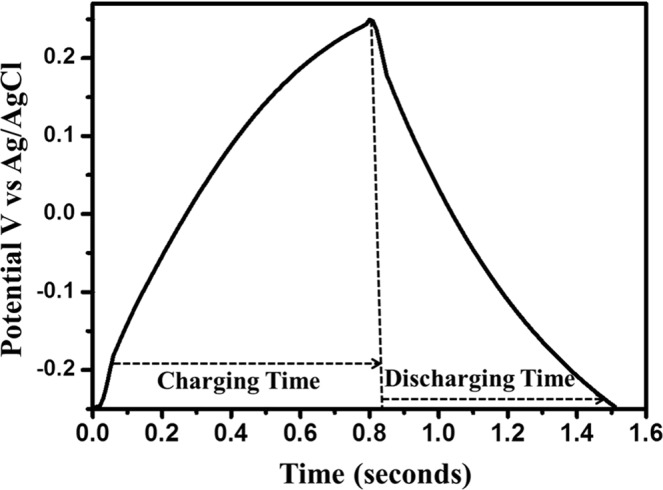
Peak current vs square root of scan rate plot of Randles-Sevcik equation to understand the diffusion controlled electrochemical nature of the as prepared electrode materials.

Under the fixed potential window and different current densities, galvanostatic charge and discharge technique was used to measure the value of electrochemical capacitance as well as the columbic efficiency at the highest current density (Fig. 12). As mentioned earlier, the potential window was fixed at −0.25 V to +0.25 V. Nearly-triangular shapes of the galvanometric curves confirm the EDLC characteristics of the samples. Current densities varied differently for different material. KHC and CHC were able to sustain a high value of current density at 5 A/g as given in Fig. 12a,c. SHC was able to sustain a very low value of 0.3 A/g. Columbic efficiency values are given based on their highest value as calculated from the percentage ratio of the discharge time to charging time of respective materials. Interestingly, the columbic efficiency of KHC has the highest value of 108.61% for 2 A/g current density and its sustainability with different current densities are higher than that of others. CHC gives a decent 93.9% columbic efficiency, which is higher than all of the rest. This makes CHC a strong candidate for supercapacitor applications along with KHC. This experimental data also lies in the same understanding line with the judgements made based on the characterization techniques. SHC and PHC also show an appreciable columbic efficiency of 91.17% and 93.97%. All the columbic efficiencies were calculated for the first cycle just for a comparison. The specific capacitance value of KHC showed a slight of 5.77% after 100 cycles and the columbic efficiency becomes 96.47% after the 100^th^ cycle, which confirms the stability and sustainability of this material over cycles. To understand the charge and discharge profile better, one triangle of the CP profile is taken as depicted in Fig. 13. The exhibited triangular behaviour implicates at EDLC capacitive nature but the visible deviation from typical linearity gives it a curvilinear shape, which is the reason behind its pesudocapacitive nature. The certain and sudden drop in current value at the immediate start of discharging is due to the internal resistance of the material.

**Figure 14:**
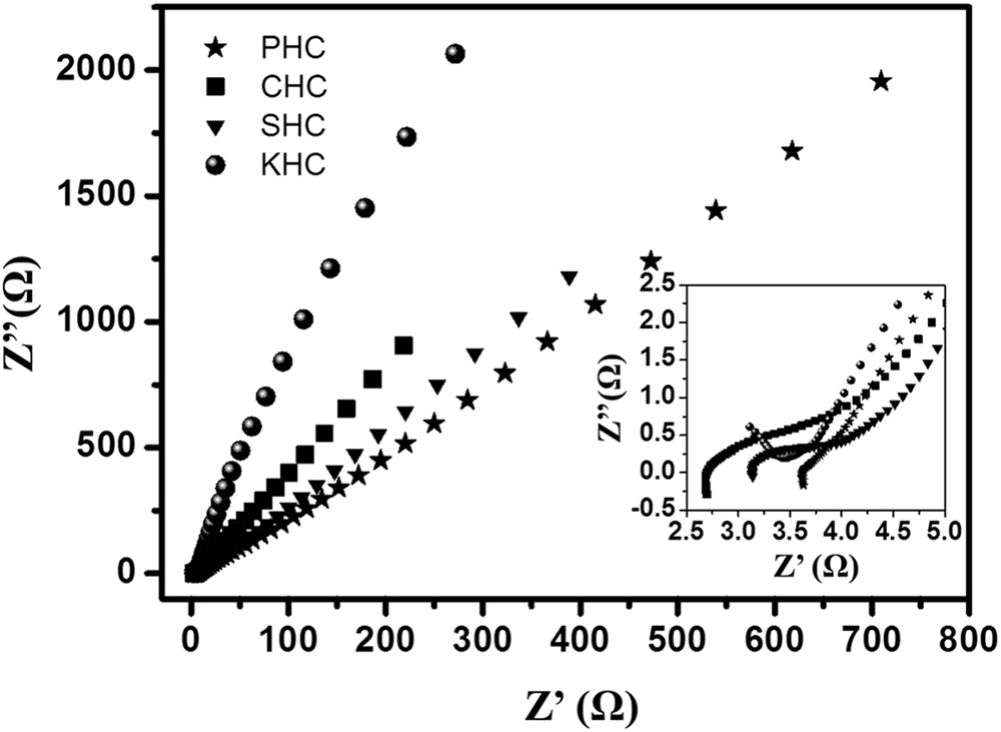
Nyquist plot of hard carbon electrodes after applyinga sine wave with amplitude of 0.005 V over the frequency range from 0.10 Hz to 0.1 GHz. Inset: Enlargement of the Nyquist plots in high frequency region.

EIS is a technique to measure the internal resistance of working electrode material and determine the circuitry and corresponding resistance between the electrolyte and the electrode, like hard carbon in this case. A sine wave of 5 mV amplitude was applied and the frequency was varied from 0.01 Hz to 0.1 GHz in the 6 M KOH aqueous solution. Ionic and electronic interactions cause the overall impedance of the supercapacitor^55^. Ionic resistance is the result of the ionic diffusion, when the ions from electrolyte move into the pores. The overall EIS curve is basically the depressed semicircle in the high frequency range and linear in the low frequency zone. The process of charge transfer induces the presence of semi-circle and the linear zone defines the diffusion zone. The larger diameter of the semi-circle is indicative of higher resistance due to the transfer of charges. That is a sign of the poor conductivity of the material. Linearity confirms the reliability of the material as a perfect and ideal capacitor^3,14,55^. From the images (Fig. 14), it is evident that SHC produces the semi-circle of highest diameter. The KHC and CHC give a curve with less circular nature. This determines the effective characteristics of these two materials as supercapacitor electrodes. In the Nyquist plot, the –Z” (imaginary part) is plotted as a function of Z’ (real part) of the impedance. The overall resistance consists of electrical resistance, the resistance due to electrolyte and the resistance for the carbon pore charge-transfer as well as the internal resistance of the material. KHC and CHC stand out among all four samples as possible supercapacitor material, where KHC provides a better value of capacitance. Activation process has increased its specific surface area and pore volume, which is a probable reason behind the capacitive performance.

**Figure 15:**
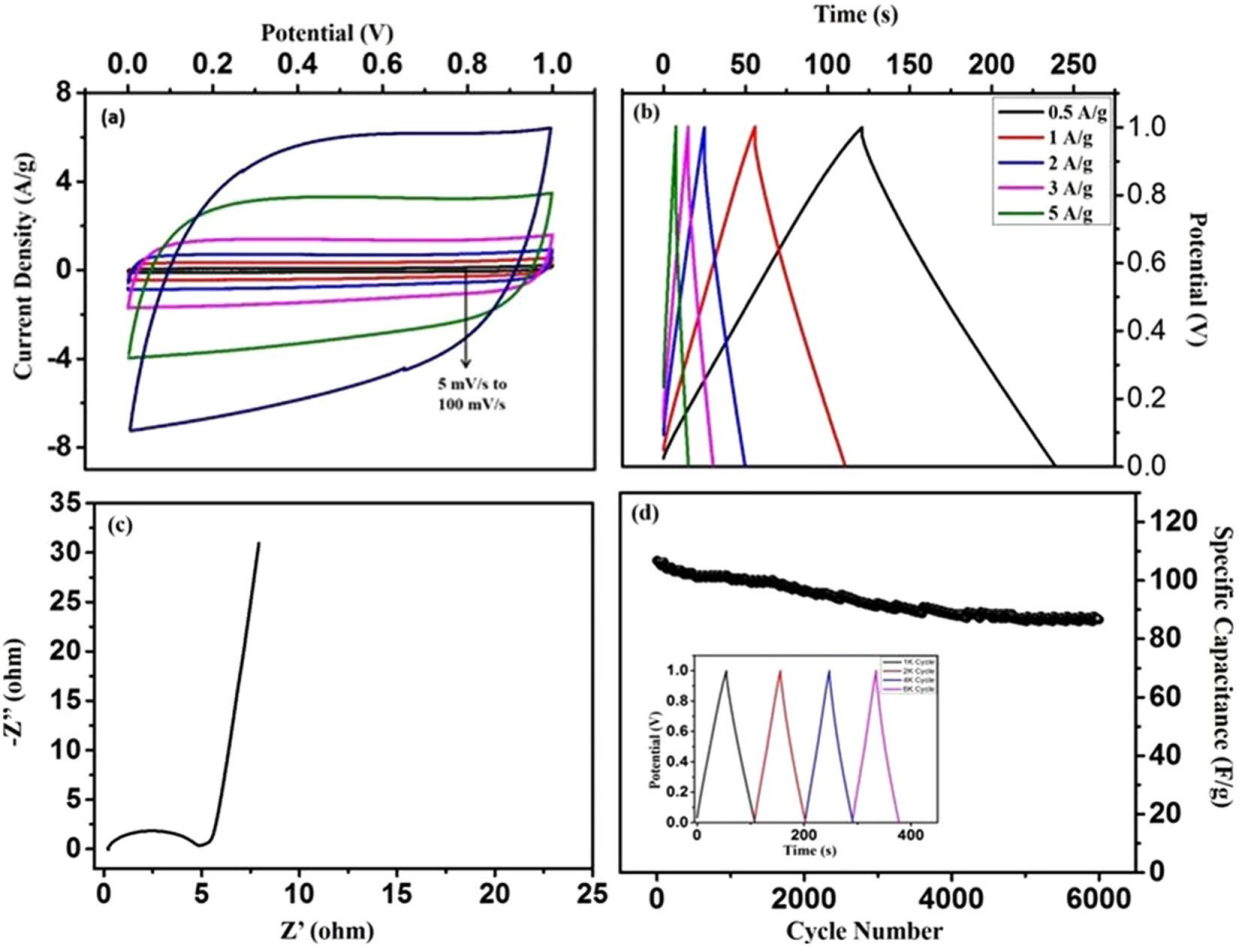
(**a**) Cyclic voltammetry of KHC based device at different scan rates from 1 mV/s to 100 mV/s, (**b**) Galvanostatic charge/discharge curves measured at various current values in 6 M KOH solution, (**c**) Nyquist plot after applying a sine wave with amplitude of 0.005 V over the frequency range from 0.10 Hz to 0.1 GHz, (**d**) Cyclic stability for over 6000 cycles in KHC.

**Figure 16:**
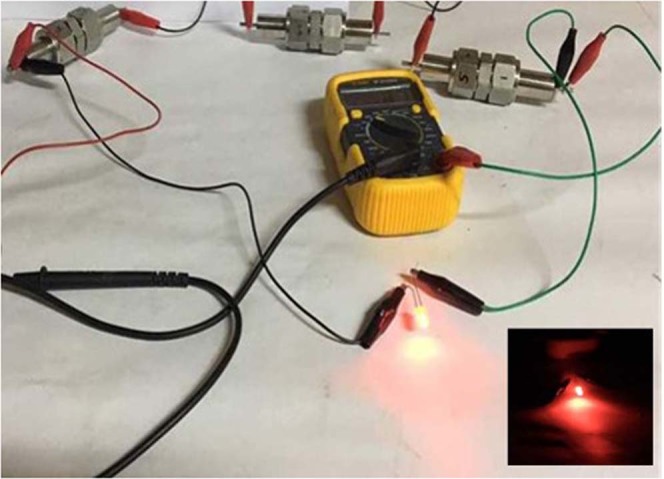
Demonstration of supercapacitor devices by using hard and activated carbon as supercapacitorelectrodes.

Figure 15a shows the CV profile at various scan rates and the material still retains a rectangular shape even at high scan rate of 100 mV/s, which indicates a good EDLC behavior. The charge discharge profile in Fig. 15b exhibits symmetric triangular behavior with less IR drop, implying a good supercapacitive behavior. The specific capacitance calculated from CD profile at 0.5 A/g current density is 118 F/g. The Nyquist plot in Fig. 15c shows the less charge transfer resistance of 4.5 ohms in the electrode material. Since cyclic stability of material is a crucial parameter for practical application of supercapacitor, the cyclability of KHC electrode material was carried out. Figure 15d shows the variation of specific capacitance as a function of cycle number at a current density of 1 A/g. Even after 6000 cycles, the electrode material still retains an efficiency of 72.88%, indicating good stability of the material. For the practical application of KHC material, a device is fabricated in aqueous 6 M KOH electrolyte and electrochemical studies have been carried out as shown in the Fig. 16.

## Conclusions

The present study targeted to achieve the synthesis of hard carbon material synthesised from biomass in order to check the potential of the material for supercapacitor applications, which will be cheap, eco-friendly and ease synthesis procedure must be easy. The present study aimed to synthesize a cost effective and eco-friendly hard carbon from biomass and determine the suitability of the material for supercapacitors applications. Four different hard carbons were synthesized from KOH activated banana stem (KHC), phosphoric acid treated banana stem derived carbons (PHC), corn-cob derived (CHC) and potato starch derived (SHC), and further tested as supercapacitor electrodes. KOH-activated hard carbon gave better performance among all and the results are supported by various characterization techniques like Raman spectroscopy, BET and TEM. After doing comparative study of these samples with literature, it is quite clear that KHC and CHC gave decent performances in the category of carbon based materials. Thus KHC and CHC have potential to be utilized in electrochemical energy storage device as supercapacitor electrodes due to its respectable performance and favourable physical properties. A cheap, eco-friendly and easy feasible procedure to prepare hard carbon for supercapacitor application was successful.

## Experimental

### Materials

Potassium hydroxide (SRL, India), phosphoric acid (H_3_PO_4_) (Sigma Aldrich), polyacrylonitrile (Sigma Aldrich), starch soluble (potato) (SDFCL, India) were purchased and were used as received. Fresh banana stem and corns were purchased from market of Vellore, Tamil Nadu, India. De-ionized water was used to prepare solution of potassium hydroxide.

### Material synthesis

#### Synthesis of KOH activated hard carbon from banana stem (KHC)

Banana stem based activated hard carbon was prepared by a three step procedure which included dehydration, porogenic stage and pyrolysis. After obtaining fresh stems from the market, it was cleaned in distilled water and cut into small pieces. The pieces were heated at 100 °C for 24 hours as a part of the dehydration process. The de-moisturised dry stem pieces were cooled down to room temperature before KOH aqueous solution (10%) was added to it. Then the aqueous mixture was dried at 110 °C for 4 days. Finally the dried and activated banana stem pieces were carbonized for 2 h at 900 °C in the presence of N_2_ gas in a tubular furnace^57,58^. The pyrolyzed carbon material was ground to obtain fine powder of hard carbon. This synthesized material is denoted as KHC.

#### Synthesis of H_3_PO_4_ activated hard carbon from banana stem (PHC) from KHC

The material was prepared exactly the same procedure like above. After dehydration process, it was cooled down to room temperature. The dried and de-moisturized stems were impregnated in phosphoric acid (H_3_PO_4_) at a ratio of 1:5 for sample and porogenic material and were dried at 110 °C for 4days. At the end of the fourth day, the paste like gelatinous black substance was annealed for 2 h at 900 °C in the presence of N_2_ gas^38,59^. The annealed and dried black hard carbon was ground to produce fine powder denoted as PHC.

#### Synthesis of corncobs derived hard carbon (CHC)

Corncobs derived hard carbon was synthesized by direct pyrolysis method as per the earlier report^47^. Corncobs were dried at 100 °C for 24 h, and after that they were carbonized for 2 h in a tubular furnace under argon gas flow at 1000 °C temperature to get the desired material, denoted as CHC.

#### Synthesis of potato starch derived hard carbon (SHC)

The synthesis of hard carbon from starch was carried out by a simple procedure which involved two steps. Primarily, soluble potato starch was heated at 200 °C. The foam, created by this preheating process was ground and heated at 1000 °C for 2 h under Ar atmosphere. The starch was heated at 200 °C to prevent the melting and foaming while carbonization was carried out at a high temperature^60^. The black carbonized material was ground to produce fine powder of hard carbon, denoted as SHC.

#### Materials characterization

The X-ray diffraction measurements were performed with Bruker D8 Advance Ruker, USA, Ni-filtered Cu Kα (1.5406 A°). The N_2_ adsorption and desorption isotherm analysis of the sample was done by Quantachrome Nova-1000 system at 77 K temperature. Brunauer–Emmett–Teller (BET) equation was used to measure and analyze the pore size of the samples and density functional theory (DFT) was used for measuring pore size distribution. Raman spectra were taken by Horiba Scientific with a green laser of wavelength 532 nm. Micro-structural analysis was done by high resolution TEM instrument, equipped with SAED from FEI Tecnai, G2 20 Twin operating at 200 KV. Morphological study and related mappings were obtained by using SEM of Zeiss, EVO 18 and EDX respectively.

#### Electrochemical testing

The electrochemical performances were studied with a CHI-660C electrochemical workstation. A three-electrode based system was obtained and potential differences were recorded from the perspective of Ag/AgCl as the reference electrode. Carbon paper was taken as the primary substrate for working electrode and platinum wire was used as counter electrode. Nafion solution was used as binder material while coating the electroactive material dispersed in ethanol, on carbon paper (5 cm × 1 cm × 0.1 cm). After coating, the electrode was dried at room temperature overnight prior to measurements with the area of the active material being 1 cm^2^. All the electrochemical performance and corresponding measurements were taken in 6 M KOH at room temperature (25 °C). The experiment was run at various scan rates from 1 mV/s to 100 mV/s in a potential window of −0.25 V to +0.25 V vs. Ag/AgCl reference electrode. Charge and discharge curves were measured in the same fixed potential window of −0.25 V to +0.25 V at various current densities. Electrochemical impedance measurements were carried out in the range of 0.01 Hz to 0.1 GHz with AC amplitude of 5 mV and a bias potential of 0.1 V. The impedance Z was expressed in terms of a real (Z’) and an imaginary (Z”) component. The equation that is used to measure specific capacitance is C_cv_ = (I_mx_-I_mn_)/mv, where *m* is the sample mass loaded per unit area in gm and *v*is the scan rate, Iis the loaded current and C_cv_ is the obtained value of specific capacitance in F/g.

## Data Availability

All data generated or analysed during this study are included in this published article.
